# Exploratory analysis of CD63 and CD203c expression in basophils from hazelnut sensitized and allergic individuals

**DOI:** 10.1186/s13601-016-0134-7

**Published:** 2016-12-13

**Authors:** Bianca Lötzsch, Sabine Dölle, Stefan Vieths, Margitta Worm

**Affiliations:** 1 Department of Dermatology and Allergology, Allergy-Center-Charité, Charité - Universitätsmedizin Berlin, Charitéplatz 1, 10117 Berlin, Germany; 2 Paul-Ehrlich-Institut, Langen, Germany

**Keywords:** Food allergy, Hazelnut, Basophil activation test, CD63, CD203

## Abstract

**Background:**

Sensitization to hazelnut (HN) is frequent and requires clarification to determine whether this sensitization is clinically relevant. The aim of this study was to investigate basophil activation profiles in HN-sensitized and allergic subjects.

**Methods:**

Basophil activation was determined by flow cytometric analyses of CD63 and CD203c expression using several HN allergen concentrations. Depending on their clinical reaction pattern, an oral allergy symptom group (OAS, n = 20), a systemic reaction group (n = 12) and a sensitized group without clinical symptoms (n = 20) were identified. Additionally, 10 non-allergic and non-sensitized individuals served as controls.

**Results:**

CD63 and CD203c expression differed between allergic (OAS and systemic group) and sensitized subjects. The HN concentration required to activate 30% of CD203c^+^ basophils [effective concentration (EC)30] was significantly higher in sensitized versus the allergic group (p = 0.0089). This was more pronounced when the basophil allergen threshold sensitivity (CD-sens) was calculated (CD63: p = 0.018; CD203c: p = 0.009).

**Conclusion:**

Our data indicate that the basophil activation test may provide information to better distinguish between sensitized and allergic subjects if several allergen concentrations are considered. CD203c expression displayed a better discrimination compared to CD63; therefore, its diagnostic value might be superior compared with CD63.

**Electronic supplementary material:**

The online version of this article (doi:10.1186/s13601-016-0134-7) contains supplementary material, which is available to authorized users.

## Background

In Europe, hazelnut (*Corylus avellana*) is a frequent cause of food allergy [1] with a prevalence of 0.1–0.5% [2]. In adults, hazelnut (HN) allergy is often associated with a pre-existing birch pollen allergy that can develop after inhalant sensitization with the major birch pollen allergen Bet v 1, a pathogenesis-related protein of family 10 [3]. Homologous immunoglobulin (Ig)E-binding epitopes of the pollen-related major HN allergen Cor a 1 are responsible for cross reactivity to Bet v 1 [4, 5]. In Central Europe, sensitization to Bet v 1 is the major cause of pollen-associated food allergies [1]. Up to 50–90% of birch-pollen-allergic patients develop sensitivity to food such as HN, apple, and others [3].

In addition to Cor a 1, other HN allergens, namely, Cor a 2 (profilin), Cor a 8 (lipid transfer protein), Cor a 9 (legumin-like protein), Cor a 11 (vicilin-like protein) and Cor a 14 (2S albumin), have been described to cause HN sensitization [6–8].

Although, HN allergy frequently leads to oral allergic symptoms (OAS), it can also cause severe and even life-threatening reactions [9, 10].

The diagnosis of food allergy mainly includes a detailed case history, skin prick testing (SPT) and the measurement of food-specific IgE (sIgE) [11, 12]. However, the low specificity of SPT and sIgE may cause over-diagnosis, which leads to unnecessary diet restrictions resulting in a lower quality of life [13]. Thus, the diagnosis of a food allergy should be proven by double-blind, placebo-controlled oral food challenges (DBPCFC) [11, 12]. Although, DBPCFC is the gold standard in diagnosing food allergies, it is not often included in daily evaluation for several reasons such as limited time and resources [14, 15].

Diagnostic tests that may support the discrimination between sensitized and symptomatic subjects without the high risk of developing an anaphylactic reaction are desirable. The basophil activation test (BAT) is an in vitro test that determines the expression of defined basophil markers (CD63 and CD203c) after allergen activation. The BAT has been suggested as a useful tool for the diagnosis of different IgE-mediated allergies [16].

The aim of this study was to investigate the CD63 and CD203c activation profiles in sensitized and symptomatic HN-allergic subjects considering several HN allergen concentrations. Moreover, we correlated the data from these surface markers with diagnostic parameters (SPT and sIgE), which are the most frequently used.

## Methods

### Subjects

Subjects were recruited from the Allergy-Centre-Charité (Berlin, Germany). Four groups (control, sensitized, OAS, systemic) were stratified based on case history including a detailed questionnaire about their symptoms after ingesting HN and the SPT data. Symptoms like itching or swelling of the oral mucosa (lips, tongue, palate), as well as throat tightness and dysphagia were counted for OAS. The following symptoms were considered as systemic: dyspnea, vomiting, emesis, diarrhea, generalised urticaria, general erythema, angioedema, as well as cardio-vascular symptoms, and rhinitis/rhinoconjunctivitis. Exclusion criteria were pregnancy, lactation, the use of antihistamines, and immunomodulating or immunosuppressive drugs. The study was approved by the local ethics committee of Charité (EA-No.: 1832/Si.258). All subjects gave written informed consent to participate in the study.

### Skin prick test (SPT)

A SPT was performed (according to the recommendations [17]) with birch extract (Alk-Abelló, Wedel, Germany) and with fresh food (HN, apple, celery, carrot) by using the prick-to-prick method. Histamine dihydrochloride (10 mg/ml, ALK-Abelló) and sodium chloride (0.9% NaCl, ALK-Abelló) served as positive and negative controls. SPT was considered positive if the wheal diameter was ≥3 mm after 15 min.

### Total and specific immunoglobulin E

The serum samples were stored at −20 °C until use. The measurement of the total and sIgE to rBet v 1, rCor a 1.04 (hereinafter referred to as rCor a 1) and rCor a 8 were determined with the ImmunoCAP System (Thermo Fisher Scientific, Uppsala, Sweden) according to the manufacturer’s instructions. In addition, Cor a 9, Cor a 11 and Cor a 14 were measured by the Paul-Ehrlich-Institute (Langen, Germany).

### Basophil activation test (BAT)

For measurement of basophil activation, heparinized blood samples were taken from subjects and analyzed within 24 h. The blood samples were stimulated with increasing concentrations (10^−6^–10 µg/ml) of HN extract (European hazelnut extract, Greer Labs, Lenoir, NC, USA) and incubated for 15 min at 37 °C. Anti-human IgE (BIOZOL HP6029/HP6061, Eching, Germany) was used as positive, and RPMI 1640 medium (Bio Chrom AG, Berlin, Germany) was used as a negative control. Cells were stained with anti-CD63-FITC (BD Biosciences, Franklin Lakes, NJ, USA), anti-CD203c-PE (Immunotech Inc., Canada), anti-CD3-VioBlue (Myltenyi Biotec GmbH, Bergisch-Gladbach, Germany) and anti-CCR3-APC (R&D Systems, Minneapolis, MN, USA). After adding lysing solution (BD Biosciences, Franklin Lakes, NJ, USA), the basophils were analyzed by flow cytometry (Miltenyi MACS Quant Flow Cytometer, Bergisch-Gladbach, Germany). CD3^−^/CCR3^+^ cells were gated as basophils, CD3^−^/CCR3^+^/CD203^high+^ cells were analyzed for basophil activation, and CD3^−^/CCR3^+^/CD63^+^ cells were defined as degranulated basophils. The data were analyzed using the MACSQuantify Software™ program (Miltenyi Biotec GmbH). Expression values were normalized for basophil activation induced by anti-IgE. The analysis of CD203c expression was based on the allergen concentration, which activated 30% of CD203c^+^ basophils [effective concentration (EC)30] as previously performed [18]. For CD63, the maximal percentage of CD63 up-regulation at one allergen dose (CD-max) was measured. Additionally, the basophil allergen threshold sensitivity (CD-sens) and EC50 (the effective concentration giving 50% of maximum up-regulation) were calculated as described by Glaumann et al. [19]. Subjects with less than 15% change in CD3^−^/CCR3^+^/CD203^high+^ or CD3^−^/CCR3^+^/CD63^+^ expression between negative and positive control were regarded as non-responders and excluded from further analysis (n = 5).

### Statistical analysis

Statistical analysis was performed with IBM SPSS Statistics (Chicago, IL, USA) and Prism (GraphPad Software, San Diego, CA, USA). All results are shown as medians with ranges (min–max, unless otherwise stated). Differences between the groups were verified with the Kruskal–Wallis test or Mann–Whitney U test for non-Gaussian distribution. The Spearman correlation coefficient was used for comparison between the diagnostic tests. p values <0.05 were considered to be statistically significant. Significant results should be regarded as descriptive and explorative due to the small sample size in this study.

## Results

### Identification of subjects

**Fig. 1:**
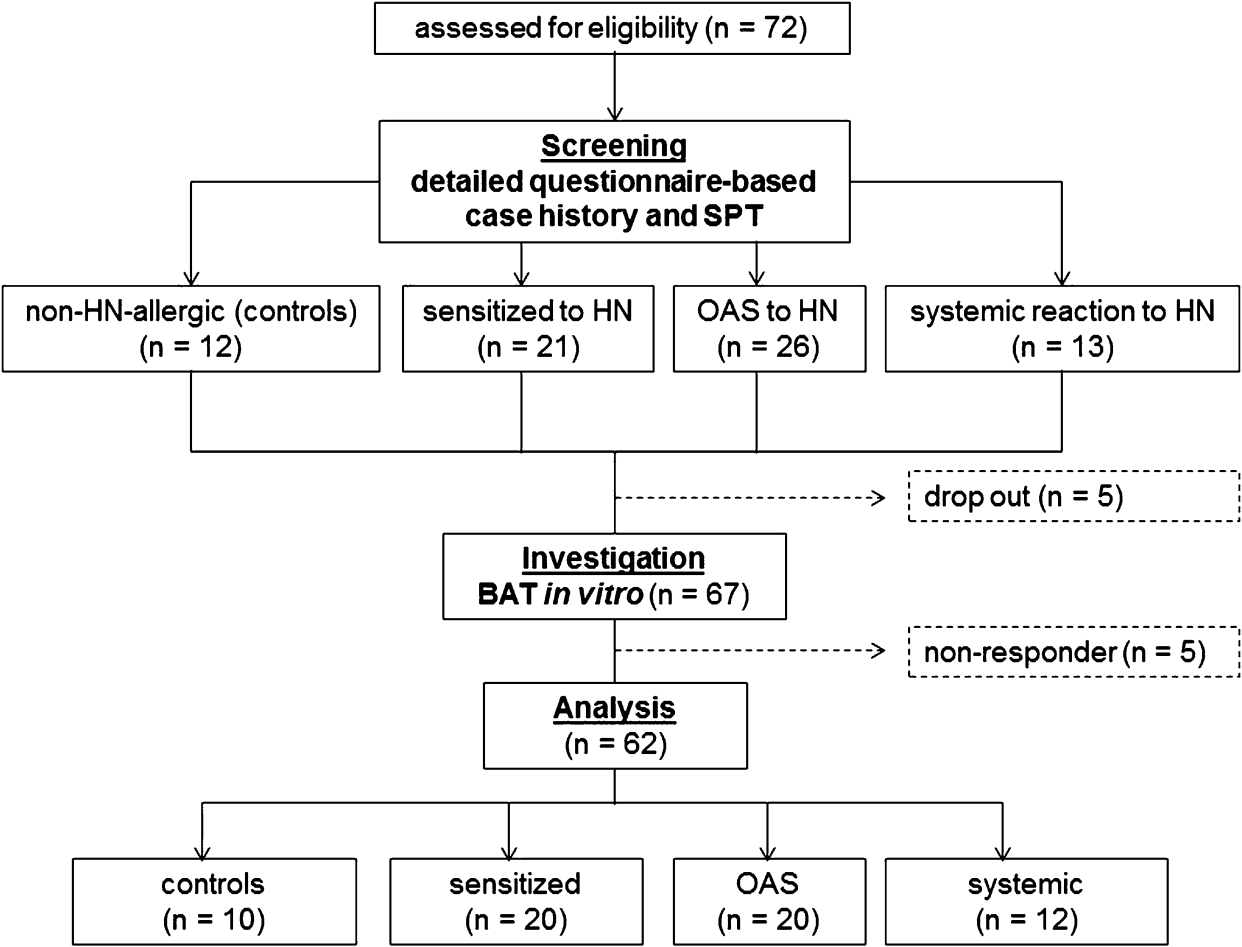
Study flow chart and classification of subjects. *HN* hazelnut, *SPT* skin prick test, *OAS* oral allergic symptoms, *BAT* basophil activation test

In total, 72 subjects were screened, and the BAT was performed in 67 individuals; of these, 62 were analyzed [median age: 31 (19–62) years, n = 40 (65%) females and n = 22 (35%) males; Fig. 1; Table 1]. Table 1 summarizes the subjects’ characteristics. In this study, 10 subjects had a negative SPT for HN and birch and no symptoms when consuming HN (controls) and 20 subjects showed no symptoms but had a positive SPT for HN (sensitized). In addition, 32 subjects had a positive SPT for HN and according to their clinical symptoms, these were divided into subjects with OAS (n = 20) and subjects with systemic reactions (n = 12).

**Table 1:** Subjects’ characteristics

	Controls (n = 10)	Sensitized (n = 20)	OAS (n = 20)	Systemic (n = 12)	p values
Age (median [min–max] in years)	30 [20–57]	26 [19–60]	32 [21–51]	40 [24–62]	NS
Male sex (%)	2 (20%)	12 (60%)	6 (30%)	2 (17%)	0.041^c^
Atopic history [number (%)]					
Allergic rhinitis	3^a^ (30%)	14 (70%)	16 (75%)	9 (75%)	
Atopic dermatitis	2 (20%)	5 (25%)	9 (50%)	5 (33%)	
Allergic asthma	–	9 (35%)	10 (55%)	8 (58%)	
SPT (median [min–max] in mm)					
Birch	0*	6 [0–12]	7 [0–11]	9 [3–11]	NS
Hazelnut	0*	4 [3–8]	5 [3–10]	6 [4–14]	NS
Celery	0*	0 [0–7]	3 [0–10]	4 [0–7]	NS
Apple	0*	3 [0–8]	4 [0–7]	5 [0–20]	NS
Carrot	0*	0 [0–10]	3 [0–15]	4 [0–14]	NS
IgE (median [min–max] in kU/l)					
Total-IgE	16 [3–34]*	184 [20–2097]	178 [24–6861]	59 [26–4801]	NS
sIgE Bet v 1	0.0 [0.0–0.1]*	3.5 [0.0–16.2]	10.2 [0.0–72.4]	5.9 [0.1–43.6]	NS
sIgE Cor a 1	0.0 [0.0–0.1]*	1.9 [0.0–7.4]	6.7 [0.0–63.8]	3.5 [0.1–28.1]	0.0254^d^
sIgE Cor a 8	n.d.	n.d.	0.2 [0.0–1.3]	0.0 [0.0–0.0]	NS
Ratio^b^ (median [min–max])					
rBet v 1/total-IgE	0.00*	0.04 [0.00–4.19]	0.07 [0.00–13.0]	0.04 [0.00–11.9]	NS
rCor a 1/total-IgE	0.00*	0.02 [0.00–0.80]	0.20 [0.00–4.36]	0.18 [0.00–4.77]	0.0226^e^

Statistical significances were analyzed with Kruskal–Wallis test

* Significant difference of the control group versus the other groups

^a^According to case history, not caused by birch pollen

^b^Calculated as sIgE * sIgE/total IgE

^c^Sensitized versus control and systemic group

^d^Sensitized versus OAS group

^e^Sensitized versus OAS and systemic group

### Sensitization profile

SPT and sIgE were used to assess the sensitization status. All subjects from the sensitized, OAS and systemic groups had a positive SPT to HN (inclusion criteria). The wheal diameters for HN were not significantly different between the sensitized and symptomatic groups (Table 1). A similar pattern was observed when the birch pollen extract was used. In the symptomatic group, all 32 subjects, and in the sensitized group, 15 of 20 subjects, had a positive SPT to birch pollen extract (Table 1).

Total IgE levels were comparable among the sensitized, OAS and systemic groups and as expected, significantly higher compared to the control group (Table 1). In the sensitized group, the sIgE level against rCor a 1 [1.9 (0.0–7.4) kU/l] was significantly lower compared to the sIgE level from the OAS group [6.7 (0.0–63.8) kU/l], but not compared to the systemic group [3.5 (0.1–28.1 kU/l)]. For both symptomatic groups, higher ratios of sIgE to total IgE levels were calculated for Cor a 1 in comparison to the sensitized group. The ratios for rBet v 1 were comparable between the sensitized and symptomatic groups (Table 1). No sensitization to Cor a 9, Cor a 11 or Cor a 14 were found.

### Basophil activation profiles

The BAT was performed in 67 screened subjects; among these, 5 (3.35%) were non-responders (Fig. 1).

**Fig. 2:**
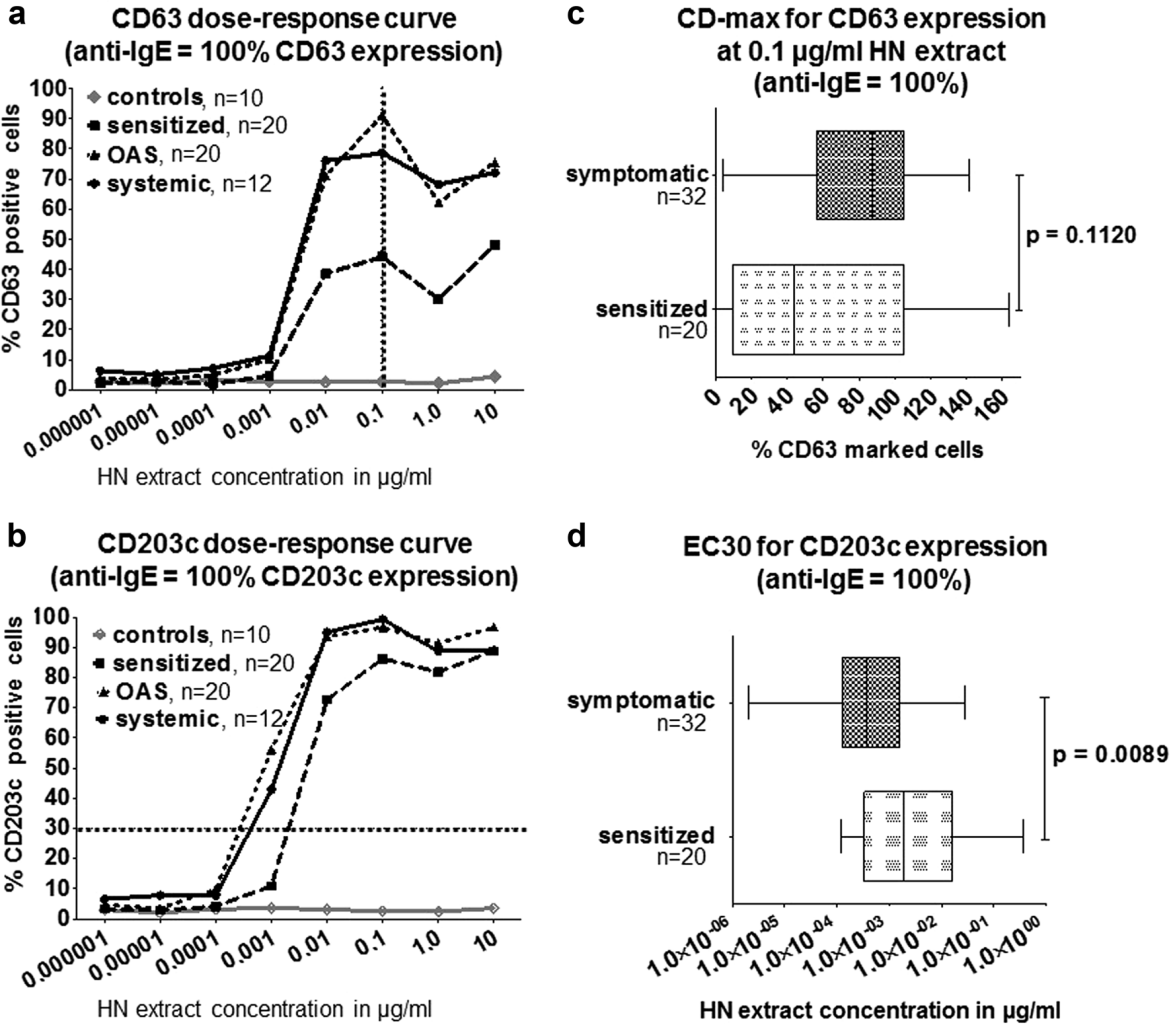
Expression of CD63 and CD203c normalized for basophil activation induced by the positive control (anti-IgE). **a**, **b** The dose–response curves for CD63 (**a**) and CD203c (**b**). The values are shown as medians. For a clear picture, the ranges are not shown in **a** and **b**. The net values are depicted in Additional file 1: Figure S1 as medians with interquartile ranges for the four groups. The *vertical dotted line* indicates the maximum CD63 expression (CD-max) at the hazelnut (HN) concentration of 0.1 µg/ml (**a**), and the *horizontal dotted line* gives the concentration required to activate 30% of CD203c^+^ basophils [effective concentration (EC)30] (**b**). **c**, **d** The comparison between the sensitized (n = 20) and symptomatic (OAS and systemic, n = 32) groups by **c** Maximum CD63 expression (CD-max) at 0.1 μg/ml of hazelnut (HN) extract and **d** HN extract concentrations that activated 30% of basophils (EC30). Statistical significances were analyzed with Mann–Whitney U test

The CD63 expression measured as basophil reactivity (maximal reactivity, CD-max) showed differences between clinically symptomatic (OAS/systemic) and sensitized subjects (Fig. 2a). The median CD63 expression in the sensitized group was lower for all HN extract concentrations than the median of CD63 expression of the subjects with symptomatic HN allergy. However, no significant differences were detected by comparing CD-max, which was measured at a concentration of 0.1 μg/ml of HN extract in the groups (sensitized, OAS, systemic) (Fig. 2c). Additionally, EC50 and CD-sens for CD63 were calculated (Table 2). We detected a significant higher CD-sens in the symptomatic group: 44.73 (13.01–96.72; p = 0.018) compared to the sensitized group: 6.59 (0.07–57.84). However, we could not differentiate between the OAS and systemic groups.

**Table 2:** Basophil allergen threshold sensitivity (CD-sens) and half-maximal effective concentration (EC50)

Subject group	No. of subjects	CD63			CD203c		
		EC50 (ng/ml)	CD-sens	p values	EC50 (ng/ml)	CD-sens	p values
Sensitized	20	15.18 (1.73–1336)	6.59 (0.07–57.84)		10.09 (1.21–33.38)	10.03 (3.55–82.90)	
OAS	20	2.24 (1.05–7.12)	44.73 (14.07–96.38)	0.028	0.97 (0.17–6.95)	104.55 (14.92–602.48)	0.006
Systemic	12	2.61 (0.98–9.94)	40.40 (10.23–104.61)	NS	1.74 (0.55–7.95)	59.35 (13.27–190.12)	NS
Symptomatic (OAS + systemic)	32	2.24 (1.04–7.70)	44.73 (13.01–96.72)	0.018	1.27 (0.37–7.02)	80.33 (14.64–267.87)	0.009

Values are given as median with interquartile range (IQR). Statistical significances were analyzed with Mann–Whitney U test compared to the sensitized group

For CD203c, the effective concentration by which 30% of basophils were activated (EC30) was calculated. Higher HN concentrations were required to achieve an increase of CD203c expression in sensitized subjects (Fig. 2b), which was significant when calculated for EC30 (p = 0.0089, Fig. 2d). In order to better compare, we calculated EC50 and CD-sens for CD203c too (Table 2). The measured differences between CD-sens of the symptomatic group: 80.33 (14.64–267.87) and the sensitized group: 10.03 (3.55–82.90) were significant (p = 0.009). Again, we could not differentiate between the OAS and systemic group.

### Correlations between SPT, sIgE and BAT

We identified statistical correlations (p < 0.001) between the CD63 and CD203c expression values at single HN concentration versus SPT, sIgE to rCor a 1, and sIgE to rBet v 1. The highest correlation was detected for 0.01 µg/ml and is summarized in Table 3. A correlation between CD-sens versus the other diagnostic measurements was only detected for CD203c (Table 3).

**Table 3:** Correlation of basophil activation test (BAT) with different diagnostic tests

	Expression values at 0.01 µg/ml HN		CD-sens	
	CD63	CD203c	CD63	CD203c
…with SPT (HN)	0.561; p < 0.001	0.620; p < 0.001	0.064; NS	0.389; p = 0.003
…with IgE values				
Total-IgE	0.203; p = 0.113	0.245; NS	−0.262; NS	−0.020; NS
sIgE rBet v 1	0.689; p < 0.001	0.777; p < 0.001	0.203; NS	0.539; p < 0.001
sIgE rCor a 1	0.690; p < 0.001	0.754; p < 0.001	0.208; NS	0.541; p < 0.001

Correlation between CD63 and CD203c expression values at 0.01 µg/ml: 0.784; p < 0.001 and CD-sens: 0.561; p < 0.001

Spearman correlation coefficient (r; p value) of BAT values at 0.01 µg/ml HN concentration and CD-sens for CD63 and CD203c with skin prick test (SPT) for hazelnut (HN), total and sIgE levels

## Discussion

Our results show that the BAT is a useful tool to determine biological activity against a food allergen such as HN. The correlation between CD203c/CD63 expression and the sensitization results measured by sIgE and SPT indicates basophil activity can be linked to sensitization status. Moreover, the CD203c-based BAT may distinguish between sensitized and symptomatic individuals but only if a careful dose–response analysis is performed.

The literature is controversial about the advantages and disadvantages of basophil surface markers [20]. CD63, compared to CD203c, is not a basophil-specific marker and has been shown to be less specific and less sensitive (in grass pollen and house dust mite allergy, latex allergy, wasp venom hypersensitivity, peanut allergy) [21–23]. On the other hand, the up-regulation of CD63 expression has been reported to provide similar results to CD203c expression in cat-allergic subjects [24] and to be more sensitive and specific in egg-allergic children [23]. All these data suggest a high variability of sensitivity and specificity depending on the allergen and cohort that was studied.

In this study, measuring CD203c expression was superior to CD63 when sensitized and symptomatic groups were considered (Fig. 2c, d; Table 2). However, with the BAT, we were not able to distinguish a patient with systemic reactions from a patient with OAS. The ability to differentiate between the symptom severities was limited either by the diagnostic assessment, which was based on clinical history, or by the Cor-a-1-based HN allergy in our cohort. It is known that Cor a 1 can induce severe allergic symptoms but may require the intake of larger amounts of the Bet v 1 homologues such as it is the case for Gly m 4 in soy-containing dietary food products, or it may need allergen-protective matrix effects [12]. The biological activity of Cor a 1 might be very high in birch pollen endemic regions, [25] thus, the difference between OAS and more severe allergic reactions are not detected in the BAT.

CD203c as well as CD63 expression values at a HN concentration of 0.01 µg/ml correlated with the SPT and sIgE results (Table 3). Considering CD-sens, a correlation was detected for CD203c but not CD63. Thus, the BAT can be used as a functional assay to detect sensitization status, which had been shown previously [26]. However, in our hands, CD203c seems to be superior compared to CD63. Moreover, the BAT has the potential to more closely resemble the clinical phenotype of patients [27]. In previous studies, we have shown the usefulness of CD203c expression in demonstrating differences of the allergenicity between native and roasted HN extracts [2] and between two different tomato cultivars [18]. Previous reports, where CD63 and CD203c expression were analyzed in combination, support our findings [28–30]. The data of Wanich et al. [28] indicate less basophil activity in tolerant versus symptomatic cow’s milk-allergic subjects. The BAT could also differentiate between tolerant and symptomatic peanut-sensitized subjects [30]. Santos et al. [29] demonstrated the utility of basophils as biomarkers for severity and threshold of allergic reaction in a pediatric cohort suffering from peanut allergy. Whether a combined analysis is suitable is a matter of discussion due to different expression kinetics [16, 31].

Differences regarding kinetics might be a reason for the findings when comparing CD63 and CD203c in our cohort. The maximal up-regulation of CD63 occurs within 25–30 min, whereas CD203c requires only 10–20 min [32]. The stimulation time used in this study averaged 15 min to capture both markers.

For practical reasons, basophil activation experiments should be restricted to a single allergen concentration [16, 32]. However, an individual, highly heterogeneous basophil response has been described previously [33] and was also present in our cohort. Thus, a single allergen concentration is not sufficient to analyze basophil responses.

The BAT offers many advantages in food allergy. In contrast to sIgE, the BAT provides a biological readout [34]. A wide range of food, raw material, purified or recombinant allergens can be analyzed [35]. Unspecific positive reactions are less frequent in BAT compared to SPT. In addition, SPT bears the risk of sensitization [36]. Moreover, basophils can be analyzed even when the subject receives anti-allergic treatment [37]. However, other studies demonstrated an inhibitory effect of immunosuppressants such as cyclosporin A [38] or other drugs (statins) [39]. Therefore, a careful history regarding a possible drug intake is mandatory if a BAT is considered.

The diagnosis of food allergy is mainly based on patient history, analysis of IgE and/or SPT, ideally combined with DBPCFC, which is still the gold standard in the diagnosis of food allergy [11, 12]. However, DBPCFC is expensive and time-consuming for both the physician and the patient and is often refused by the patient. Additionally, there is a risk for patients to experience severe allergic reactions. Thus, in this study, a detailed case history including allergy-focused diet history assessment was used to define the status of HN allergy, which had previously been shown to have a high diagnostic values [40, 41].

However, an ex vivo but highly diagnostic method to reliably predict clinical reactivity is desirable. Neither the presence of sIgE in the circulation nor the presence of biologically active IgE on mast cells are suitable to differentiate between sensitization and clinical allergy [35], with perhaps the exception of Cor a 9 and Cor a 14 in children cohorts [41, 42]. For both allergens, an age-dependent variability seems to exist, as allergic adults are less sensitized to both components [43]. In our cohort, no sensitization to Cor a 9, Cor a 11 or Cor a 14 was found. Thus, in our cohort Cor a 1 is the predominate allergen. The sIgE to Cor a 1 was lower in the sensitized group compared to the symptomatic groups (significant for OAS group), which was more pronounced when calculated as a sIgE/total-IgE-ratio. Glaumann et al. [19] investigated the utility of BAT in an Ara h 8-sensitized cohort. Ara h 8 is a Bet v 1 homologue in peanut like Cor a 1 in HN. They could show that the BAT adds safety information if sIgE and DBPCFC results were controversial. Thus, the BAT might be useful if clinical history, SPT and/or sIgE measurement are inconsistent or in addition an oral food challenge is not possible [42]. Furthermore, the use of recombinant allergens in the BAT might have added value in discriminating between clinically relevant and mere sensitization [44, 45] and should be considered in future studies.

Most BAT protocols are not standardized. For the future, protocol optimization is required and should consider preanalytical conditions and well-defined flow cytometry gating protocols [46]. The timeframe of analysis is relevant as well; ideally, the BAT should be performed immediately after a blood draw [37, 46] but at least within 24 h, as longer storage time can lead to a loss of basophil reactivity and false negative results [37].

A weakness of this study is the small number of analyzed subjects and the results should be regarded explorative. However, we were able to differentiate between non-allergic, sensitized and symptomatic subjects. The inclusion of HN-sensitized subjects is a strength of the study, as such a group is essential to validate a diagnostic test in allergy [33]. Still, due to the small sample size, necessary diagnostic parameters (sensitivity, specificity, positive and negative predictive value) were not calculated. Furthermore, the diagnosis of a symptomatic HN allergy was based on clinical history, as mentioned above. Moreover, we did not prove that the activation of the basophils with the HN extract was exclusively via cross-linking surface-bound sIgE against HN (Cor a 1) or via cross-reaction of surface-bound sIgE against birch homologues (Bet v 1).

The sensitivity and specificity of the BAT is influenced by the rate of non-responders. These patients do not show activation after anti-IgE stimulation [33]. The reason for this non-responsiveness of the basophils is a selective decrease in Syk expression, which is a downstream event of FcεRI activation [33]. The proportion of non-responders depends on the BAT protocol and varies between 5 and 25% [32, 33]. In this study, 3.25% of patients when considering both expression markers and up to 6.5% of patients when considering only CD63 expression were non-responders.

## Conclusion

In summary, the CD203c-based BAT differentiates between sensitization and clinically relevant allergy in HN-sensitized individuals. The use of a given allergen extract at several concentrations is required, and the development of standardized protocols are needed to compare results obtained from different cohorts and allergens.

### Additional file

**Additional file 1: Figure S1.** Dose–response curves for CD203c (*left*) and CD63 (*right*) expression for the four different groups (**a–d**). Net values without normalization to anti-IgE are shown as median with interquartile range (IQR). (+) anti-IgE (−) medium.

## Abbreviations

BAT: basophil activation test
CD-max: the maximal percentage of surface marker up-regulation at one allergen dose
CD-sens: basophil allergen threshold sensitivity
DBPCFC: double-blind, placebo-controlled oral food challenges
EC: effective concentration
HN: hazelnut
IgE: immunoglobulin E
sIgE: specific immunoglobulin E
OAS: oral allergy symptom
SPT: skin prick test
