# Supplementary figures and images for: Exploratory analysis of CD63 and CD203c expression in basophils from hazelnut sensitized and allergic individuals
Source: Clin Transl Allergy. 2016 Dec 13;6:45. doi: 10.1186/s13601-016-0134-7 (PMC5153676; doi:10.1186/s13601-016-0134-7)

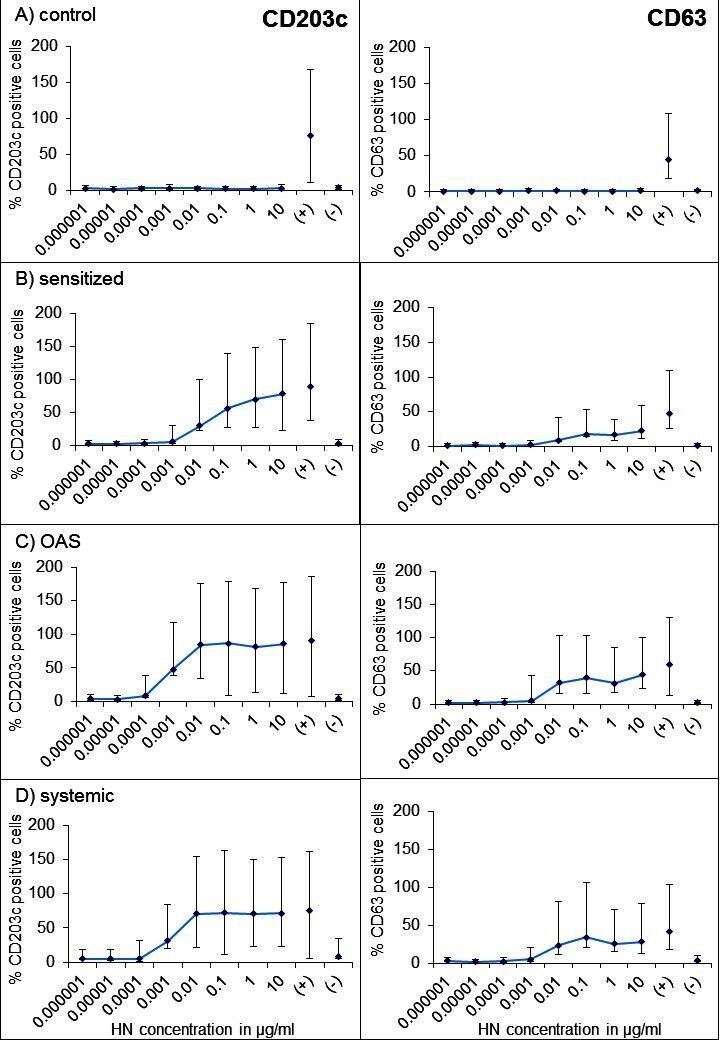

Supplement: Supplementary file 1 — Additional file 1: Figure S1. Dose–response curves for CD203c (left) and CD63 (right) expression for the four different groups (a–d). Net values without normalization to anti-IgE are shown as median with interquartile range (IQR). (+) anti-IgE (−) medium. [file 13601_2016_134_MOESM1_ESM.jpg]
